# PReoperative very low-Energy diets for obese PAtients undergoing non-bariatric surgery Randomized Evaluation (PREPARE): a protocol for a pilot randomized controlled trial

**DOI:** 10.1186/s40814-024-01511-6

**Published:** 2024-05-21

**Authors:** Tyler McKechnie, Karim Ramji, Maisa Saddik, Jordan Leitch, Ameer Farooq, Sunil Patel, Aristithes Doumouras, Sameer Parpia, Cagla Eskicioglu, Mohit Bhandari

**Affiliations:** 1 Department of Surgery, Division of General Surgery, McMaster University, 1280 Main St. W, Hamilton, ON L8S 4L8 Canada; 2 Department of Health Research Methods, Evidence, and Impact, McMaster University, Hamilton, ON Canada; 3 Department of Surgery, Division of General Surgery, St. Joseph Healthcare, Hamilton, ON Canada; 4 Department of Anesthesia and Perioperative Medicine, Queen’s University, Kingston Health Sciences Centre, Kingston, ON Canada; 5 Department of Surgery, Division of General Surgery, Queen’s University, Kingston Health Sciences Centre, Kingston, ON Canada; 6 Department of Oncology, McMaster University, Hamilton, ON Canada; 7 Department of Surgery, Division of Orthopedic Surgery, McMaster University, Hamilton, ON Canada

**Keywords:** Surgical procedures, Obesity, Weight loss, Weight loss products, Preoperative optimization, Prehabilitation

## Abstract

**Background:**

Patients with obesity presenting in need of surgical intervention are at 2-to-sixfold higher risk of prolonged hospitalization, infectious morbidity, venous thromboembolism, and more. To mitigate some of these concerns, prescribed preoperative weight loss via very low-energy diets (VLEDs) has become a standard of care for patients with obesity undergoing bariatric surgery. While VLEDs have become standard prior to bariatric surgery, their application in other surgical settings remains limited. A large, definitive trial is required to resolve the uncertainty surrounding their use in these patients. Prior to a definitive trial to compare the efficacy of VLEDs in patients with obesity undergoing major non-bariatric surgery, we require a pilot trial. We argue a pilot trial will provide the following critical feasibility insights: (1) assessment of recruitment ability, (2) evaluation of adherence to VLED regimens, and (3) assessment of our ability follow patients completely.

**Methods:**

The proposed trial will be a multi-center, surgeon, outcome assessor, and data-analyst blinded, parallel pilot randomized controlled trial (RCT). Patients older than 18 years of age with a body mass index (BMI) of greater than 30 kg/m^2^ undergoing major elective non-bariatric surgery will be eligible for inclusion. Consecutive patients will be allocated 1:1 according to a computer-generated randomization schedule. Randomization will be stratified by center and will employ randomly permutated blocks. All patients in the intervention group will receive standard patient counseling on weight loss and an active VLED protocol. The preoperative VLED protocol will utilize commercially available weight loss products for three weeks preoperatively. The primary outcomes (randomization percentage, recruitment rate, intervention adherence, follow-up completion, network development) will assess feasibility. Descriptive statistics will be used to characterize the study sample.

**Discussion:**

The PREPARE pilot RCT will aim to provide feasibility and safety data that will allow for the successful completion of the definitive PREPARE trial that has the potential to provide practice changing data pertaining to the regular use of VLEDs as a means of pre-habilitation for patients with obesity undergoing major non-bariatric surgery.

**Trial registration:**

This study was registered on ClinicalTrials.gov (reference #NCT05918471) on June 23, 2023.

**Supplementary Information:**

The online version contains supplementary material available at 10.1186/s40814-024-01511-6.

## Introduction

Obesity is a worldwide epidemic affecting upwards of 700 million people [1]. The economic burden in the United States and Canada are estimated at over $2 trillion and $100 billion United States dollars (USD), respectively, and the resultant healthcare consequences can be devastating for systems and patients alike [2]. Obesity is a systemic disease with significant consequences for patients undergoing any form of medical or surgical intervention [3, 4]. Patients with obesity presenting in need of medical and surgical intervention are at 2-to-sixfold higher risk of prolonged hospitalization, infectious morbidity, venous thromboembolism, and more [5–8]. In the postoperative period in particular, their risk of morbidity is significantly increased. Incidence of cardiovascular (1–2% vs. 2–4%), genitourinary (3–10% vs. 6–20%), and wound (3-–9% vs. 6–18%) complications are at least doubled compared with patients without obesity [9–13]. These worsened short-term postoperative outcomes not only prolong recovery following surgery, but they also result in significantly longer length of stay and greater healthcare spending [14, 15].

To pre-emptively mitigate some of these concerns, prescribed preoperative weight loss via very low-energy diets (VLEDs) has become a standard of care for patients with obesity undergoing bariatric surgery (i.e., weight loss surgery) [16]. This type of surgery typically involves restricting oral intake or bypassing parts of the gastrointestinal tract to limit macronutrient absorption to help patients lose weight. The Canadian Adult Obesity Clinical Practice Guidelines and Enhanced Recovery After Surgery (ERAS) guidelines recommend 2–3 weeks of preoperative VLED with liquid formulations prior to bariatric surgery [16, 17]. These programs can effectively induce significant amounts of preoperative weight loss [18–20]. This contributes to decreased postoperative length of stay (LOS) by 0.5–1 days and decreased fat content by as much as 29% around the major organs resulting in better visualization and improved ease of the surgery [20–23]. The impact on postoperative morbidity, however, is less clear. We performed a systematic review and meta-analysis of four small RCTs (sample sizes 20 to 273) evaluating VLEDs in obese patients undergoing bariatric surgery demonstrating a 33% reduction in the risk of major complications with VLED—a point estimate suggesting an important benefit; however, the wide 95% confidence intervals (CIs) and resultant type II error risk create significant uncertainty (RR 0.67, 95% CI 0.39–1.17, *p* = 0.16, *I*^2^ = 0%) [18].

While VLEDs have become a standard in patients with obesity undergoing bariatric surgery, largely due to the significant reduction of peri-organ fat for better visualization, their application to obese patients undergoing other types of surgery remains limited [24]. We performed a systematic review and meta-analysis that identified 13 studies evaluating the use of preoperative VLEDs in patients with obesity undergoing orthopedic, vascular, colorectal, upper gastrointestinal, gynecological, and a variety of general surgery procedures for benign disease (e.g., cholecystectomy, abdominal wall hernia) [24]. While data were heterogenous, preoperative VLEDs reliably resulted in significant weight loss (3.2–19.2 kg) with high rates of adherence to the protocols (94–100%). Adverse event rates were low (< 14% in most studies). There were no significant differences in postoperative outcomes, though again pooled analyses included a small number of patients and there were wide corresponding 95% CIs. We have also shown that other forms of preoperative weight loss, such as bariatric surgery, prior to major abdominal surgery can improve postoperative outcomes [25]. Specifically, weight loss induced by bariatric surgery prior to surgery for colorectal cancer can contribute to a 25% relative risk reduction in overall postoperative morbidity, 43% relative risk reduction in gastrointestinal morbidity, and 47% relative risk reduction in respiratory morbidity [25]. Preoperative weight loss by way of preoperative VLEDs has the potential to do the same [20]. The evidence for routine use of VLEDs in patients with obesity undergoing major non-bariatric surgery is compromised by heterogeneous, small studies with methodological limitations. A large, definitive trial is required to resolve this uncertainty. Prior to a definitive trial to compare the efficacy of VLEDs in patients with obesity undergoing major non-bariatric surgery, we require a pilot RCT. We argue a pilot trial will provide the following critical feasibility insights: (1) assessment of recruitment ability, (2) evaluation of adherence to VLED regimens, and (3) assessment of our ability follow patients completely.

## Materials and methods

### Pilot trial objectives

The objective of this pilot RCT is to determine the feasibility of a multicenter RCT comparing patients with obesity (i.e., BMI > 30 kg/m^2^) receiving VLEDs versus control prior to elective non-bariatric surgery in terms of perioperative outcomes. The specific feasibility objectives of this trial are as follows: (1) determine the feasibility of recruiting patients in a timely manner across local and outside sites; (2) determine adherence with preoperative VLEDs in patients with obesity undergoing elective non-bariatric surgery; (3) determine the feasibility of completion of follow-up; (4) determine our ability to develop a network of participating sites in a multi-site initiative; (5) determine the safety of administering preoperative VLEDs to patients with obesity undergoing elective non-bariatric surgery. We postulate this pilot RCT will demonstrate feasibility and thus offer support for a full RCT aimed at evaluating the efficacy of preoperative VLEDs in patients undergoing major non-bariatric surgery in terms of perioperative outcomes.

### Trial design

The proposed trial will be a multi-center, surgeon, outcome assessor, and data-analyst blinded, parallel pilot RCT. The trial will be reported according to the Consolidated Standards of Reporting Trials (CONSORT) Extension for Randomized Pilot and Feasibility Trials [26].

### Study centers

This pilot trial will include 4 centers: (1) St. Joseph’s Healthcare Hamilton (local); (2) Juravinski Hospital, Hamilton (local); (3) Hamilton General Hospital (local); (4) Kingston Health Sciences (outside site). Throughout the pilot RCT, we will be seeking active engagement from at least 6 other centers to have at least 10 centers involved in the full RCT.

### Trial population

The inclusion criteria are as follows: (1) older than 18 years of age, (2) BMI of greater than 30 kg/m^2^, (3) undergoing major elective non-bariatric surgery*.* Major surgery is defined as any thoracic, abdominal, cardiovascular, orthopedic, oncologic, or reconstructive operation performed under general anesthesia requiring a skin incision extending beyond the subcutaneous tissue.

The exclusion criteria are as follows: (1) undergoing bariatric surgery; (2) undergoing neurologic surgery; (3) undergoing urgent or emergent surgery; (4) recently diagnosed myocardial infarction or unstable angina (i.e., within past 6 months); (5) diagnosed moderate-to-severe renal dysfunction (i.e., eGFR less than 30 mL/min/1.73 m^2^); (6) diagnosed severe liver dysfunction (i.e., cirrhosis, portal hypertension, hepatic encephalopathy, hepatorenal syndrome); (7) recently diagnosed alcohol or drug use disorders (i.e., excessive use of substance within past 6 months); (8) recently diagnosed, uncontrolled eating disorder (e.g., bulimia nervosa, binge eating disorder within the past 12 months); (9) recent episode of gout (i.e., within past 6 months); (10) history of porphyria; (11) known allergy to any Optifast® or Medimeal® ingredient; (12) enrolled in other prospective studies with similar interventions and/or outcomes; co-enrollment may be deemed appropriate if the steering committees of the respective trials review the details of participation and determine the likelihood of interaction between interventions is low; (13) pregnant or breastfeeding women; (14) patients residing in a long-term care facility; (15) patients unable to provide informed consent.

### Recruitment

Patient recruitment will occur at Hamilton Health Sciences (i.e., Juravinski Hospital, Hamilton and Hamilton General Hospital), St. Joseph’s Healthcare Hamilton, and Kingston Health Sciences. All surgeons who perform elective non-bariatric surgery at these sites will be made aware of the study via workshops and internal communications. Patients will be identified by the surgeons, their administrative assistants, patient navigators, and/or study investigators at the time of referral or at the time of their initial preoperative consultation. Surgeons and/or other clinicians within a patient’s circle of care will introduce the study to participants at the time of their preoperative visits. Patients meeting inclusion criteria based on a review of their electronic medical record will be approached by designated research personnel at the time of their initial surgical consultation in clinic. If the patient is not identified in time, or if it is not feasible for the research personnel to approach the patient in person at the time of their consultation, the patient will be contacted by telephone within one-week of their consultation. At the time of initial contact between the study personnel and patient, the study will be introduced, and eligibility will be confirmed. Once eligibility is confirmed, the written informed consent process will ensue. After documentation of written informed consent, patients will be registered using a web-based randomization system. Consecutive patients will be allocated 1:1 according to a computer-generated randomization schedule to one of the two treatment arms. Randomization will be stratified by center and will employ randomly permutated blocks.

### Planned trial interventions

#### Intervention group (VLED protocol plus counseling)

All patients in the intervention group will receive patient counseling on weight loss and an active VLED protocol. The preoperative VLED protocol will utilize commercially available weight loss products, such as Optifast® 900, Medimeal®, or a similar product. Patients will receive a 3-week supply. They will start taking the VLED 3 weeks and 2 days prior to their surgery date (i.e., 23 days prior to their surgery date) and finish the intervention the evening of their second last day prior to surgery. This timeframe was chosen to not interfere with any other perioperative intervention required for a given surgery (e.g., bowel preparation, carbohydrate loading). They will be instructed to consume four packets daily. This provides a total energy intake of 900 kcal. Patients will also be able to consume up to 2 cups of low-calorie vegetables per day along with the meal replacement product. They will be provided with a handout containing specific instructions. Patients will keep self-report diaries of their dietary intake and activity levels. Patients enrolled in the trial and assigned to the VLED group who are diabetics will be referred to a diabetes educator nurse practitioner for management of their insulin dosing and/or other anti-hyperglycemic medications throughout the duration of the intervention period.

The current protocol in use at our local Bariatric Surgery Center utilizes four doses commercially available VLED daily and the duration varies between 1 and 3 weeks based on liver size and BMI. We have adapted this for the purposes of the present trial as presented in Additional file 1. To standardize the approach for non-bariatric surgery, we propose an intervention period of 3 weeks. In our systematic review of preoperative VLEDs prior to non-bariatric surgery, 95% CIs for weight loss resulting from programs lasting between 3 and 8 weeks overlapped significantly [24]. This plateau coupled with time constraints for the preoperative waiting period for certain patient populations (e.g., cancer patients), served as reasoning for our 3-week intervention period. Our systematic review identified protocols varying from 0.46 to 26 weeks that were safe and efficacious, and thus we are confident that our intervention period is appropriate [24]. In our systematic review and meta-analysis, Optifast® was the most commonly studied VLED liquid formulation (35.7% of included studies) and daily target caloric intake ranged from 450 to 1400 kcal per day [24].

Adherence to a preoperative 2-to-3-week regimen of preoperative VLED prior to bariatric surgery ranges from 80 to 90% [27, 28]. In our systematic review and meta-analysis evaluating the use of VLEDs in non-bariatric surgery, reported adherence ranged from 94 to 100% among the included studies, with much longer durations of intervention (0.46–24 weeks) [24]. We anticipate adherence in this study will be similar and range between 80 and 100%. To increase adherence with the study medication, we will cover the cost of the VLED and have patients complete a diet diary.

#### Control group (counseling alone)

The control group patients will receive the pre-existing standard of preoperative care, which may include counseling for weight loss without prescription of a specific preoperative weight loss intervention, as this is meant to be a pragmatic trial. Currently, there are no standardized interventions aimed at optimizing obese patients prior to undergoing non-bariatric surgery. Briefly, counseling may consist of the surgeon, at the time of the preoperative clinic visit, discussing weight loss strategies such as decreased caloric intake and increased physical activity. Patients will not receive prescriptions for preoperative VLEDs, any other weight loss supplement, or any physical activity intervention aimed at promoting weight loss prior to surgery. Patients will keep self-report diaries of their dietary intake and activity levels.

#### Perioperative care for all study participants

Given the pragmatic nature of this trial, all patients will receive surgical care as per their respective surgeons. Dosing of preoperative antibiotics and venous thromboembolism prophylaxis will be performed for all included patients where appropriate. Intraoperative care and procedures, including anesthesia, will be determined as per the independent practitioners and the type of surgery. Patients will be enrolled in ERAS programs for postoperative recovery where appropriate.

### Outcomes

Detailed criteria for event adjudication are reported in Additional file 2. A blinded outcome adjudication committee will independently identify and report outcomes. This committee will be blinded to treatment allocation. Given that this is a pilot trial, the primary outcome will be feasibility.

#### Feasibility outcomes

1. Randomization percentage: Defined as the number of patients agreeing to participate in the RCT and being randomized to treatment or control divided by the number of patients approached for participation in the RCT. A randomization percentage of 70% or greater will support the feasibility of a full RCT. A lesser randomization percentage may be feasible with modifications.
2. Recruitment rate: Defined as the number of patients randomized into the RCT per month. We will aim for a rate of 16 patients per month (i.e., 4 patients at each of 4 sites per month). A recruitment rate of equal to or greater than this will support the feasibility of a full RCT. A lesser recruitment rate may be feasible with additional sites for the full RCT. At present, we anticipate participation of at least 10 sites in the definitive trial. If each site recruited at least 4 patients per month, this would equate to a minimum 48 patients per year at a given site. Thus, recruitment over 2.5 years would achieve our anticipated study sample size of approximately 1200.
3. Intervention adherence: Defined as the number of preoperative VLED doses taken divided by the total number of doses prescribed for each participant randomized to the intervention arm. Adherence will be self-reported via written diaries. A mean adherence of greater than 80% (i.e., completing 80% or more of doses) will be our benchmark for feasibility in the present study. Adherence will also be measured through matching actual preoperative weight loss with expected preoperative weight loss. As per our systematic review evaluating preoperative VLEDs in patients undergoing bariatric surgery, patients receiving preoperative VLEDs for 2–3 weeks prior to bariatric surgery, they loss approximately 5% of their pre-VLED weight [18]. Therefore, a study participants expected weight loss will be defined as 5% of their pre-VLED in kilograms. We will subtract their expected weight loss from their actual weight loss as a measure of adherence.
4. Follow-up completion: Defined as completion of the pre-VLED, post-VLED, and 30-day postoperative visits, along with complete anthropometric measures and study questionnaires. A follow-up completion rate of greater than 90% will support the feasibility of a full RCT.
5. Network development: Defined as recruiting from all four participating centers at the aforementioned rate. Additionally, we will aim to extend our multi-disciplinary network to at least 10 centers throughout the course of this pilot RCT in preparation for the full RCT.
6. Other findings: Unexpected and/or findings unique to this pilot trial will be reported narratively.

#### Safety outcomes

The primary safety outcome will be all adverse events deemed secondary to the preoperative VLED. The adverse events will be recorded as dichotomous outcomes and described as either minor or serious, similarly to the OPTIWIN study: the largest medical weight loss RCT evaluating VLEDs (*n* = 273) [29]. Standardized definitions for adverse events are described in Additional file 3 (adjudicated outcome).

#### Clinical outcomes

The COMET initiative was consulted to assess for a pertinent core outcome set (COS) [30]. After reviewing the database, no COS for preoperative weight loss was identified. The efficacy outcomes include those in which preoperative weight loss could have a plausible effect:

1. Overall 30-day postoperative morbidity (primary): This will be defined as any deviation from the usual postoperative course within 30 days of the index operation and will be a composite of system-specific complications (Additional file 2) (adjudicated outcome)
2. 30-day system-specific complications (secondary) (adjudicated outcome)
3. 30-day postoperative mortality (secondary) (adjudicated outcome)
4. Preoperative weight loss (secondary): Preoperative weight loss will be assessed by measuring the post-VLED weight on the date of surgery and adjusting for the baseline weight in kilograms
5. Operative time (secondary): Operative time will be measured as the time between first skin incision and closure of the last surgical wound in minutes and will be retrieved from the patient EMRs
6. Intraoperative blood loss (secondary): This will be measured in milliliters and collected from the patient chart
7. Intraoperative complication (secondary): This will be defined as any documented complication in the operative note from the index procedure that aligns with any one of the following: hemorrhage: more than 1000 mL of intraoperative blood loss and/or receipt of a blood transfusion[31], iatrogenic injury to nearby organ/structure, conversion to open procedure
8. Postoperative LOS (secondary): This will be measured in days and collected from the patient chart
9. Surgeon-perceived difficulty (secondary): Surgeon-perceived difficulty will be evaluated with a short electronic questionnaire administered immediately following completion of the case. Questionnaires for non-bariatric abdominal and orthopedic surgery are currently being created and validated by the present research team.
10. Quality of life (secondary): This will be assessed at baseline, following completion of the VLED, and 30 days postoperatively using the Short-Form 36 (SF-36). The SF-36 has been validated in previous cohorts of patients undergoing non-bariatric surgery [32, 33].

### Follow-up schedule

Patients will be followed at baseline, following completion of their VLED prior to surgery, and 30 days following their index surgery date (Table 1).

**Table 1:** Standard Protocol Items: Recommendations for Interventional Trials (SPIRIT) figure for participant timeline

Activity	Before randomization	− 23	After randomization			
	** − 60 to − 23**		** − 23 to − 2**	**0**	**0 to discharge**	** + 30**
Eligibility assessment	X					
Informed consent	X					
Randomization		X				
Intervention (i.e., VLED)			X			
Baseline demographic data	X					
Operative data collection				X		
Baseline anthropometrics	X					
Post-intervention anthropometrics				X		
Post-surgical anthropometrics						X
Intervention-associated adverse events			X	X	X	X
Postoperative morbidity					X	X
Surgeon perceived difficulty				X		
QoL assessment (i.e., SF-36)	X			X		X
Intervention compliance			X	X		
Follow-up completion						X

*VLED*, very low-energy diet; *QoL*, quality of life

#### Baseline

Demographic data will be collected as well as anthropometric measures. Number of patients approached, number of patients consented, and number of patients declined, as well as reasons patients declined, will be recorded to assess feasibility. QoL data will be ascertained via administration of an in-person paper SF-36 questionnaire by a blinded research assistant.

#### Post-VLED completion

Patient-reported compliance will be assessed via a patient-completed diet diary that they will complete daily during the 3-week period prior to surgery. Both the treatment and control arms will complete these forms and give them to the research assistant in an opaque envelope on the date of their surgery. Patients in the control arms will be instructed to leave the VLED component of the form blank. Anthropometric measures will take place through a blinded research assistant. VLED-related safety data will be ascertained via a combination of patient self-reporting and EMR data. Paper questionnaires will be used to assess the presence of adverse events. QoL data will be ascertained via administration of an SF-36 questionnaire. Intraoperative difficulty will be assess using a short survey immediately following completion of the operation.

#### Thirty days postoperatively

Postoperative outcomes will be ascertained via review of the EMR. They will be reviewed on the date of surgery and at 30 days postoperatively. We will assess operative time, intraoperative blood loss, and postoperative LOS via EMRs. Anthropometric data will be measured. QoL data will be ascertained via administration of an SF-36 questionnaire. The number of patients completing the 30-day postoperative follow-up will be recorded to assess feasibility.

### Sample size calculation

We propose a pilot RCT of 88 patients (i.e., 22 patients per site) to assess the feasibility of a full RCT aimed at determining the efficacy of preoperative VLED at improving short-term postoperative outcomes in obese patients undergoing non-bariatric surgery. This sample size is sufficient to ascertain our feasibility objectives and is approximately 7.5% of the size of a definitive trial. A full RCT would require 1158 patients (Additional file 4). We justified our proposed sample size of 88 patients based on 95% CIs for one of our feasibility outcomes. Specifically, to assess for 90% follow-up completion, 88 patients would provide 95% CIs of 82–95%, which we believe is adequate precision.

The anticipated recruitment rate is 16 patients/month. Given that patient enrollment will continue to 88, we anticipate recruitment will be complete within 6 months. A review of the 2018–2019 St. Joseph’s Healthcare Hamilton database including patients undergoing non-bariatric abdominal surgery, plastic surgery, otolaryngology surgery, and orthopedic surgery suggests 300–400 elective non-bariatric surgeries/month. A review of local retrospective studies suggests that 40–50% of patients undergoing non-bariatric surgery are obese (i.e., BMI > 30 kg/m^2^). Therefore, there will be approximately 120–160 potentially eligible patients per month. Across four sites, this equates to approximately 480–640 patients/month that may be eligible [34, 35]. Thus, a total recruitment of 16 patients/month is a plausible estimate across four sites.

### Analysis strategy

Descriptive statistics will be used to characterize the study sample. The outcomes of the pilot study will be descriptive in nature and will focus on feasibility. Differences in binary outcomes will be presented using absolute differences and corresponding 95% CIs. Similarly, differences in continuous outcomes will be presented as mean differences with 95% CIs. We will not conduct hypothesis testing for outcomes [36]. We will calculate aggregated measures for 30-day postoperative morbidity to compare this to the power calculation for the full RCT, to ensure an accurate power calculation is performed for the full RCT. There will not be a planned interim analysis in the present study. There are no subgroup analyses planned [37].

### Trial management

We have assembled a committed community of surgeons and solid trial infrastructure to pursue this endeavor and execute a large, definitive RCT aimed at assessing the efficacy of preoperative VLEDs for patients with obesity undergoing major non-bariatric surgery. The committee structure for this research program is as follows.

#### Central coordinating and methods center

The Clinical Trials Group, Department of Surgery at McMaster Methods Center, experienced in large trials, will coordinate this trial. Trials successfully designed and executed by this coordinating center include the following: FLOW (2345 patients, NEJM), FAITH (1100 patients, Lancet), and HEALTH (1400 patients, NEJM). This group has the infrastructure in place to successfully conduct high-impact, large RCTs with large numbers of research coordinators, data managers, statisticians, and investigators. The Clinical Trials Group will be responsible for the daily conduct of the trial, including randomization, data management, adjudication, and data analysis.

#### Data management

Data management will be overseen by Dr. Sameer Parpia, a PhD biostatistician with significant experience in managing data for large RCTs.

#### Steering committee

Interdisciplinary experts in the field with significant methodological experience and expertise (Drs. Mohit Bhandari, Sameer Parpia, Cagla Eskicioglu, Aristithes Doumouras) will comprise the steering committee. The steering committee will oversee design, data collection, data management, interpretation of the data, and manuscript generation. This committee will hold the primary responsibility for publication of the trial results. The project officer (i.e., Dr. Tyler McKechnie) will meet with the steering committee at least once during the planning, recruitment, and close-out phases of the trial, and at any other time point as deemed necessary by the principal investigators.

#### Data monitoring committee (DMC)

Our DMC will be comprised of 3 members who remain completely independent of the study investigators and have never received any honoraria from, or held stock, in any of the companies whose products are used in this trial. The DMC members will include a clinical expert with prior trial experience, a clinical trial methodologist, and a biostatistician. The role of the DMC in the present study will be to review the study protocol in detail to provide suggestions on study design. Moreover, if a safety and/or privacy concern is raised by the steering committee throughout the course of this pilot trial, the DMC will review the pertinent data and subsequently provide recommendations. These terms of reference and functions are derived from the principles established by the Data Monitoring Committees: Lessons, Ethics, Statistics (DAMOCLES) Study Group charter. They have been approved by ethics committees and implemented successfully in several trials.

#### Central outcomes adjudication committee (CAC)

Our CAC will be formed with the guidance of our expert co-investigators Drs. Mohit Bhandari and Sameer Parpia. The CAC will be blinded and oversee data collection and event adjudication.

#### Equity, diversity, and inclusion (EDI) committee

This will be formed to ensure that all relevant EDI aspects of this trial are managed.

### Protocol amendments

All protocol amendments will be submitted to the Research Ethics Board (REB) as modifications prior to implementation. Approved amendments will be uploaded to the ClinicalTrials.gov trial registration page. Amendments will also be communicated during dissemination to both academic and lay audiences.

## Discussion

Obesity is increasingly prevalent in Western society [38]. There are over 90 million individuals living with obesity in the United States alone, with over half of the population projected to be obese by 2030 [39]. Since 1985, there has been over a 450% increase in the proportion of adults living with obesity [40]. This problem is set to increase as over 10% of Canadian children and adolescents are living with obesity [41]. The proportion of patients with obesity undergoing non-bariatric surgery ranges from 13 to 70%. As such, the surgical patient with obesity is unavoidable. This highlights the importance in developing robust preoperative optimization pathways for this increasingly prevalent patient population that rests on high-quality, level I evidence. The PREPARE RCT aims to respond to this need by providing level I evidence supporting the use of preoperative weight loss with VLEDs for patients with obesity undergoing major non-bariatric surgery. Prior to proceeding with a definitive RCT aimed at evaluating the efficacy of this intervention however, it is prudent to demonstrate the feasibility of this trial design and intervention.

Ultimately, the feasibility and safety results of this pilot RCT will be used to inform the design and implementation of a full RCT aimed at assessing the efficacy of VLEDs at improving perioperative outcomes for patients with obesity undergoing major non-bariatric surgery. Specifically, whether randomization percentage, recruitment rate, intervention adherence, and follow-up completion support the feasibility of a definitive RCT with or without modifications will be determined in this pilot RCT. One of the unique aspects of this pilot RCT will be the measure of adherence, which will rely on both patient-reported data as well as objective measures to assess adherence (i.e., percentage weight loss). This may serve as a benchmark for adherence measures for trials evaluating lifestyle interventions moving forward should it correlate well with patient-reported data. The inclusion of an outside center (i.e., Kingston Health Sciences) will also ensure that this methodology can be appropriately scaled to allow the successful conduct of a large, multi-center definitive RCT. Moreover, we will use this pilot RCT as a platform to build further awareness about this omnipresent issue in contemporary surgery and to recruit physicians from other centers to participate in our full RCT.

This research program comes at a pivotal time for the pre-operative care landscape. Preoperative optimization programs, otherwise known as pre-habilitation programs, are appearing with increasing prevalence along with evidence supporting their implementation. However, the majority of pre-habilitation data to date pertain to the old, frail patients undergoing surgery [42, 43]. This has become the minority of our patient population. Rather, the obesity epidemic has been in full effect for the past several decades, and as a result the surgical patient with obesity is unavoidable. The development of pre-habilitation programs aimed at improving perioperative outcomes for the surgical patient population with obesity are paramount. The PREPARE pilot RCT will aim to provide feasibility and safety data that will allow for the successful completion of the definitive PREPARE trial that has the potential to provide practice changing data pertaining to the regular use of VLEDs as a means of pre-habilitation for patients with obesity undergoing major non-bariatric surgery.

### Supplementary Information

Additional file 1. Very low energy diet instructions.

Additional file 2. Trial outcome definitions.

Additional file 3. Adverse events.

Additional file 4. Full trial power calculation.

Additional file 5. SPIRIT 2013 Checklist: Recommended items to address in a clinical trial protocol and related documents.

## Data Availability

Data sharing is not applicable to this article as no datasets were generated or analyzed during the current study.

## Abbreviations

BMI: Body mass index
CAC: Central outcomes adjudication committee
DAMOCLES: Data Monitoring Committees: Lessons, Ethics, Statistics
DMC: Data monitoring committee
DSMB: Data safety monitoring
EDI: Equity, diversity, and inclusion
EMR: Electronic medical record
eGFR: Estimated glomerular filtration rate
LOS: Length of stay
MCID: Minimally clinical important difference
PREPARE: PReoperative very low-Energy diets for obese PAtients undergoing non-bariatric surgery Randomized Evaluation
QoL: Quality of life
RCT: Randomized controlled trial
REB: Research Ethics Board
SF-36: 36-Item Short Form Survey
SPIRIT: Standard Protocol Items: Recommendations for Interventional Trials
VLED: Very low-energy diet
