# Supplementary material for: PReoperative very low-Energy diets for obese PAtients undergoing non-bariatric surgery Randomized Evaluation (PREPARE): a protocol for a pilot randomized controlled trial
Source: Pilot Feasibility Stud. 2024 May 21;10:82. doi: 10.1186/s40814-024-01511-6 (PMC11106982; doi:10.1186/s40814-024-01511-6)
Supplement: Supplementary file 1 — Additional file 1. Very low energy diet instructions. [file 40814_2024_1511_MOESM1_ESM.docx]

**
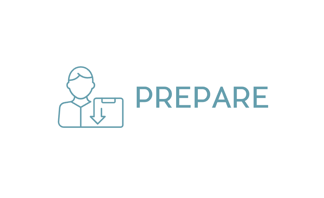
**

**PR**eoperative very low **E**nergy diets for obese **PA**tients undergoing non-bariatric surgery: A **R**andomized **E**valuation

(**PREPARE Pilot)**

*Very Low Energy Diet Instructions*

**Very Low Energy Diet Instructions**

**REDUCE Trial**

**How do I take Optifast® 900?**

1. Be sure to consume 4 Optifast® 900 or Medimeal® packets each day.
2. Take 1 sachet of your Optifast® 900 or Medimeal® once in the morning (AM), once at noon, once in the early evening (PM #1), and once later in the evening (PM #2) for three-weeks starting 23 days prior to your surgery date.
3. Mix 1 sachet with at least 300 mL (1 ¼ cups) of cold water. You may add ice cubes if you’d like. Shake well or blend in a blender.

**What else can I take with my liquid formulation?**

1. Drink at least 2 L (8 cups) of any combination of the following each day in addition to the Optifast® 900 or Medimeal®:
   1. Water
   2. Decaf coffee or decaf tea (no milk or sugar added)
      1. Artificial sweetener may be added
   3. Crystal light or Mio©
   4. Low fat broth (up to 20 kcal per serving is allowed)
   5. Sugar-free Jello
2. Can consume up to a total of 500 mL (2 cups) a day of the following while taking Optifast® 900 or Medimeal®:
   1. Green peppers
   2. Broccoli
   3. Cauliflower
   4. Lettuce
   5. Spinach
   6. Celery
   7. Cabbage
   8. Cucumber
      1. *Can add up to 15 mL (1 tablespoon) of calorie-free dressing
3. You cannot consume any other solid food while consuming Optifast® 900 or Medimeal®

**What else do I need to do for this study?**

1. Complete the “Study Diet Diary” daily.
2. If you have any questions or concerns related to this study, please contact the research coordinator or principal investigator:

Principal Investigator: Dr. Tyler McKechnie

Phone number: 613-868-9442

Email: [tyler.mckechnie@medportal.ca](mailto:tyler.mckechnie@medportal.ca)

Research Coordinator: Maisa Saddik

Phone number: 905-521-2100 ext. 43791

Email: [saddim@mcmaster.ca](mailto:saddim@mcmaster.ca)
