# Supplementary material for: PReoperative very low-Energy diets for obese PAtients undergoing non-bariatric surgery Randomized Evaluation (PREPARE): a protocol for a pilot randomized controlled trial
Source: Pilot Feasibility Stud. 2024 May 21;10:82. doi: 10.1186/s40814-024-01511-6 (PMC11106982; doi:10.1186/s40814-024-01511-6)
Supplement: Supplementary file 2 — Additional file 2. Trial outcome definitions. [file 40814_2024_1511_MOESM2_ESM.docx]

**
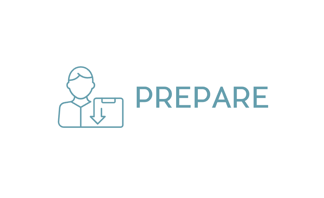
**

**PR**eoperative very low **E**nergy diets for obese **PA**tients undergoing non-bariatric surgery: A **R**andomized **E**valuation

(**PREPARE Pilot)**

*Trial Outcome Definitions*

Feasibility Outcomes

1. *Randomization percentage*

Defined as the number of patients agreeing to participate in the RCT and being randomized to treatment or control divided by the number of patients approached for participation in the RCT. A randomization percentage of 70% or greater will support the feasibility of a full RCT. A lesser randomization percentage may be feasible with modifications.

1. *Recruitment rate*

This will be defined as the number of patients recruited, enrolled, and randomized per month. Dates on which patients are approached, consented, randomized, and commence the active phase of the RCT will be recorded on an electronic form and a recruitment rate will be calculated.

1. *Optifast*® *compliance*

Defined as the number of preoperative VLED doses taken divided by the total number of doses prescribed for each participant randomized to the intervention arm. Compliance will be self-reported via written diaries. A mean compliance of greater than 80% (i.e., completing 80% or more of doses), will be our benchmark for feasibility in the present study. Compliance will also be measured through matching actual preoperative weight loss with expected preoperative weight loss. As per our systematic review evaluating preoperative VLEDs in patients undergoing bariatric surgery, patients receiving preoperative VLEDs for 2-3 weeks prior to bariatric surgery, they loss approximately 5% of their pre-VLED weight.^1^ Therefore, a study participants expected weight loss will be defined as 5% of their pre-VLED in kg. We will subtract their expected weight loss from their actual weight loss as a measure of compliance.

1. *Follow-up completion*

Defined as completion of the pre-VLED, preoperative, and 30-day postoperative visits, along with complete anthropometric measures and study questionnaires. Follow up completion percentage will be a proportion of those that successfully completed follow-up and all patients enrolled in the trial. This will be computed per trial arm as well. A follow-up completion rate of greater than 90% will support the feasibility of a full RCT.

1. *Network development*

Defined as recruiting from all three participating centers at the aforementioned rate. Additionally, we will aim to extend our multi-disciplinary network to at least 10 centers throughout the course of this pilot RCT in preparation for the full RCT.

Safety Outcomes

1. *Minor adverse events*

These will include constipation, diarrhea, nausea, fatigue, dizziness, headache, alopecia, as well as any other adverse reaction the DSMB deems as a result of the intervention. Please see Appendix B for definitions pertaining to each of the minor adverse events. Each adverse event will be adjudicated by the independent DSMB.

1. *Serious adverse events*

These will include acute kidney injury, symptomatic electrolyte disturbances, cardiac arrhythmias, symptomatic cholelithiasis, pancreatitis, pyelonephritis, gout, as well as any other adverse reaction the DSMB deems as a result of the intervention. Please see Appendix B for definitions pertaining to each of the serious adverse events. Each adverse event will be adjudicated by the independent DSMB.

Efficacy Outcomes

1. *Overall 30-day postoperative morbidity*

This will be defined as any deviation from the usual postoperative course within 30-days of the index operation and will be a composite of specific complications, including:

- Superficial incisional surgical site infection (SSI)
  - As per the Centers for Disease Control and Prevention (CDC), a surgical site infection (SSI) is “an infection that occurs after surgery in the part of the body where the surgery took place”.^2^ A sSSI will be defined as purulent drainage, tenderness, swelling, or erythema at a skin-level surgical incision.
- Deep incisional (SSI)
  - A deep incisional SSI will be defined as an SSI involving deep soft tissues of an incision (e.g., fascial layer, muscle layer) with at least one of the following:
    - Purulent drainage from a deep incision.
    - Abscess or other evidence of infection involving the deep incision on examination.
    - Spontaneous or iatrogenic fascial dehiscence with associated pathogenic organisms based on microbiologic testing and at least one of fever (>38 degrees Celsius) and/or tenderness.^2^
- Organ-space SSI
  - An organ-space infection is defined as involving any part of the surgical site deeper than the fascia/muscles with at least one of the following:
    - Purulent drainage from a drain placed into the organ-space.
    - Pathogenic organism identified on tissue or fluid culture based on microbiologic testing from within surgical organ-space.
    - Abscess or other evidence of infection involving the organ-space.^2^
- Anastomotic leak (AL)
  - Defined as per the International Study Group of Rectal Cancer as, “a defect of the intestinal wall at the anastomotic site leading to a communication between the intra- and extra-luminal compartments” as confirmed by either clinical or radiological examination.^3^ They will be graded as follows:
    - Grade A: no change in patient management
    - Grade B: requires active intervention without re-operation (e.g., percutaneous drainage)
    - Grade C: requires re-operation
- Urine leak/Urinoma
  - This will be defined based on the presence of radiographic criteria and biochemical criteria; both of the following must be present:
    - Computed tomography (CT) or ultrasound (US) evidence of an intra-abdominal or retroperitoneal collection reported by a board-certified radiologist to be in keeping with a urine leak/urinoma.
    - A drain creatinine level at least 18% greater than the plasma creatinine level.^4^
- Superficial wound dehiscence
  - This will be defined as the following finding reported on bedside clinical examination: “partial or complete separation of the layers of a surgical incision involving the skin and/or subcutaneous tissues”.^5^
- Fascial dehiscence
  - This will be defined based on the presence of either clinical criteria or radiographic criteria:^6^
    - Visible and/or palpable fascial defect at the site of the surgical incision on bedside clinical examination.
    - Evisceration of intra-abdominal contents through the surgical incision on bedside clinical examination.
    - CT evidence of disruption of the surgical incision at the level of the fascia as reported by a board-certified radiologist to be in keeping with fascial dehiscence.
  - The presence of one of these criteria will be sufficient for a diagnosis of fascial dehiscence.
- Flap necrosis
  - This will be defined based on the presence of either bedside clinical examination and intraoperative clinical examination, or microscopic tissue examination:
    - Bedside clinical examination by a surgical resident or surgeon documented as having features in keeping with flap necrosis, including any of the following:
      - Discoloration of the flap
      - Lack of capillary refill
      - Absence of bleeding with pinprick of tissue
      - Loss of sensation/pain at the site of the flap
    - Reported flap necrosis on take back to operating room for flap debridement/removal/replacement.^7^
    - Microscopic examination of flap tissue demonstrating neutrophilic infiltrate.^8^
  - The presence of the first two listed criteria or the last criteria alone will be sufficient for a diagnosis of flap necrosis.
- Prosthetic joint infection (PJI)
  - We will follow the definition proposed by the European Bone and Joint Infection Society (EBJIS).^9^ A classification of “infection likely” and “infection confirmed” will be counted as an event in the present RCT (see below).


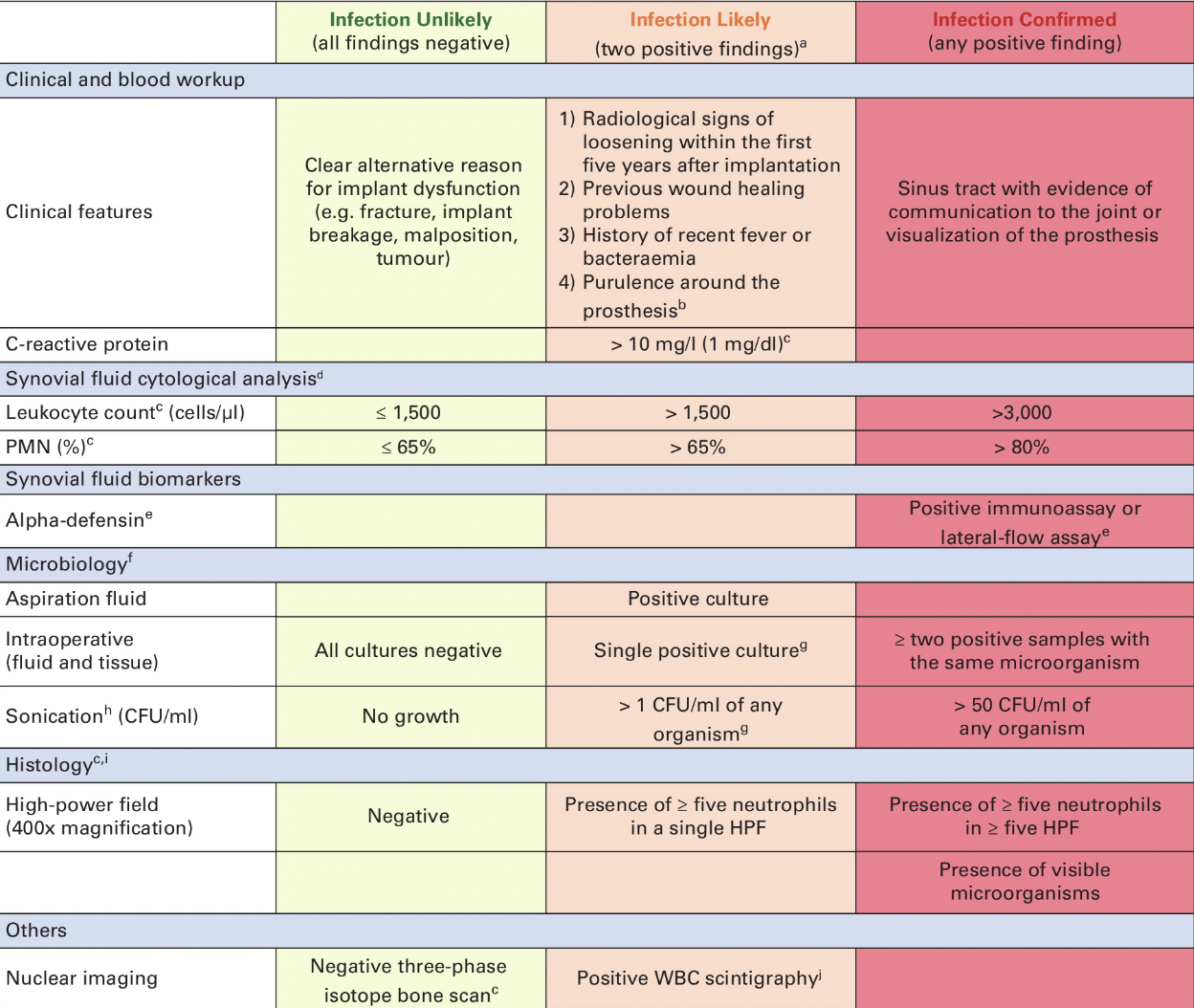


- Prolonged postoperative ileus (PPOI)
  - PPOI will be defined as, “two or more of nausea/vomiting, inability to tolerate oral diet over 24 hours, absence of flatus over 24 hours, distension, radiologic confirmation occurring on or after day 4 postoperatively without prior resolution”.^10^
- High output stoma
  - More than 1.5L of output per day for at least one day from a stoma will be defined as a high output stoma.^11,12^
- Delayed gastric emptying (DGE)
  - The International Study Group of Pancreatic Surgery defined DGE as, “the inability to return to a standard diet by the end of the first postoperative week and includes prolonged nasogastric intubation”.^13^
- Postoperative nausea and vomiting (PONV)
  - This will be defined as nausea, retching, or vomiting within 24-48 hours of anesthetic induction.^14^
- Acute urinary retention (AUR)
  - The following definition will be used for AUR: 1) inability to void within 8 hours of urinary catheter removal; or 2) difficulty voiding with a post-void residual of 400mL or greater, as measured with bedside bladder ultrasound.^15,16^
- Urinary tract infection (UTI)
  - This outcome will be defined as a urine culture with more than 10^5^ colony forming units per milliliter (mL) and clinical symptoms suggestive of a UTI.^17^
- Acute kidney injury (AKI)
  - For the purposes of this study, acute kidney injury will be defined as the presence of at least one of the following three:^18^
    - Urine output of less than 0.5mL/kg/h for more than eight consecutive hours
    - A 50-99% rise in baseline creatinine within a period of seven days
    - A decrease in eGFR of more than 25% within a period of seven days
- Atelectasis
  - New onset loss of lung volume seen on postoperative radiologic examination.^19^
- Pneumonia
  - Evidence of a new lung consolidation seen on radiologic examination along with at least one of the following:^20^
    - Signs of respiratory compromise
    - Elevated biochemical markers of inflammation (e.g., WBC, CRP)
    - Systemic inflammatory response syndrome
    - Positive sputum culture
- Pneumothorax
  - This is defined as air in the pleural cavity (i.e., between the visceral and parietal pleura) on radiologic examination or suspicion based on clinical examination and thoracic decompression with resultant release of air.^21^
- Hemothorax
  - This is defined as a collection of blood in the pleural space or a pleural fluid with hematocrit of greater than 50% obtained via thoracic decompression.^22^
- Hypoxic respiratory failure
  - This is defined as the presence of at least one of the following two clinical definitions:
    - “Hypoxia without hypercapnia and with an arterial partial pressure of oxygen (PaO₂) of <60 mmHg (<8 kPa) on room air.”^23^
    - “Presence of endotracheal intubation and positive airway pressure for at least 24 hours, along with the administration of at least 50 percent oxygen.”^24^
- Hypercapnic respiratory failure
  - This is defined as:
    - “Hypoxia with an arterial partial pressure of carbon dioxide (PaCO₂) of >50 mmHg (>6.5 kPa) on room air.”^23^
- Re-intubation
  - Re-intubation occurring within 30 days of the index operation.
- Venous thromboembolism (VTE)
  - Deep vein thrombosis (DVT) is defined as a new non-compressibility or intra-luminal filling defect of a lower or upper extremity in the deep venous system as determined by ultrasonography or venogram respectively.^25^
    - Lower extremity DVT is defined as a thrombus in the deep venous system proximal to the popliteal vessel.
    - Upper extremity DVT is defined as a thrombus in the deep venous system proximal to the axillary vessel.
  - Pulmonary embolism (PE) is defined as:^25^
    - Intraluminal filling defect in >2 views or sudden cut-off of contrast in one or more vessels >2.5mm in diameter on pulmonary angiography.
    - A high probability ventilation-perfusion lung scan in patients with high clinical probability of PE.
    - A distinct filling defect in a subsegmental or larger pulmonary vessel on helical computed tomographic scan.
    - Positive test for DVT on ultrasound or venography with a non-diagnostic ventilation/perfusion scan or a non-diagnostic helical computed tomographic scan.
- Myocardial injury after non-cardiac surgery (MINS)
  - This is defined as, “an elevated postoperative troponin measurement judged as resulting from myocardial ischemia (i.e., no evidence of non-ischaemic aetiology), during or within 30 days after non-cardiac surgery, and without the requirement of an ischaemic feature (e.g., ischaemic symptom, ischaemic electrocardiography finding)”.^26^
- Myocardial infarction (MI)
  - Any of the following meets the criteria for MI:^27^
    - Detection of rise and/or fall of cardiac biomarkers with at least one value above the 99^th^ percentile of the upper reference limit together with evidence of myocardial ischemia with at least one of the following:
      - Symptoms of ischemia
      - ECG changes indicative of new ischemia
      - Development of pathological Q waves in the ECG
      - Imaging evidence of new loss of viable myocardium or new regional wall motion abnormality
    - Sudden, unexpected cardiac death, involving cardiac arrest, often with symptoms suggestive of myocardial ischemia, and accompanied by presumably new ST elevation, or new LBBB, and/or evidence of fresh thrombus by coronary angiography and/or at autopsy, but death occurring before blood samples could be obtained, or at a time before the appearance of cardiac biomarkers in the blood
    - Pathological findings of an acute myocardial infarction
- Transient ischemic attack (TIA)
  - This will be defined based on the International Classification of Headache Disorders:^28^
    - New headache developed simultaneously with other symptoms and/or clinical signs of a TIA.
    - Resolution of the headache within 24 hours.
    - TIA most likely diagnosis based on physician opinion.
    - No radiographic abnormalities
  - The presence of at least the first three listed criteria is necessary for a diagnosis of a TIA.
- Cerebrovascular accident (CVA)
  - This is defined as, “brain, spinal cord, or retinal cell death attributable to ischemia based on:
    - Pathological, imaging, or other objective evidence of cerebral, spinal cord, or retinal focal ischemia injury in a defined vascular distribution
    - Clinical evidence of cerebral, spinal cord, or retinal focus ischemic injury based on symptoms persisting more than 24 hours or until death, and other aetiologies are excluded”^29^
- Sepsis
  - This will be recorded according to the Sepsis-3 criteria, which is based on the Sequential Organ Failure Assessment (SOFA) score, and defines sepsis sepsis as a life-threatening organ dysfunction caused by a dysregulated host response to infection. It is diagnosed when there is an increase of 2 or more points in the SOFA score from the baseline.^30^
- Surgical step-down admission
  - Admission to the surgical step-down unit within 30-days of the index operation.
- Intensive care unit (ICU) admission
  - Admission to the ICU within 30-days of the index operation.
- Reoperation
  - Take back to the operating room for diagnostic or therapeutic procedure within 30-days of the index operation.
- Readmission
  - Readmission to hospital procedure within 30-days of the index operation.

****The above will be subclassified into the following system-specific complications:*

- Infectious morbidity
  - SSI
  - PJI
  - Flap necrosis
  - Sepsis
  - UTI
  - AL
  - Pneumonia
- Respiratory morbidity
  - Atelectasis
  - Pneumonia
  - Pneumothorax
  - Hemothorax
  - Hypoxic respiratory failure
  - Hypercapnic respiratory failure
  - Re-intubation
- Cardiovascular morbidity
  - MINS
  - MI
  - TIA
  - CVA
- Gastrointestinal morbidity
  - AL
  - PPOI
  - High output stoma
  - DGE
  - PONV
- Genitourinary morbidity
  - AKI
  - UTI
  - AUR
  - Urine leak/urinoma
- Thromboembolic morbidity
  - DVT
    VTE
- Wound morbidity
  - Superficial dehiscence
  - Fascial dehiscence
  - SSI

1. *30-day postoperative mortality*

Documented death within 30-days of the index operation.

1. *Preoperative weight loss*

Preoperative weight loss is defined as the weight of the patient in kilograms the date of surgery subtracted from the baseline weight of the patient in kilograms that was taken immediately prior to commencement of the VLED. This will be measured by a blinded research nurse in a standardized fashion.

1. *Operative time*

This is the primary efficacy outcome of the study. It will be defined as the time in minutes between the first skin incision and the closure of the final abdominal wound. It is routinely recorded by bedside nurses and these data will be ascertained from the patient electronic medical chart.

1. *Intraoperative blood loss*

Intraoperative blood loss will be defined as blood lost throughout the duration of the given surgery as measured in milliliters (mL). It is routinely recorded by bedside nurses and these data will be ascertained from the patient electronic medical chart.

1. *Intraoperative complication*

Any documented complication in the operative note from the index procedure that aligns with any one of the following will be counted as an intraoperative complication:

- Hemorrhage: more than 1,000 mL of intraoperative blood loss and/or receipt of a blood transfusion.^31^
- Iatrogenic injury to nearby organ/structure.
- Conversion to open procedure.

1. *Surgeon-perceived difficulty*

Surgeon-perceived difficulty will be assessed using a short electronic questionnaire administered immediately following completion of the operation. The questionnaire is currently being developed and validated by the present research team.

1. *Quality of life*

Patient-perceived quality of life will be measured at baseline, following completion of the three-week Optifast® 900 program, and at one-month postoperatively with the Short-Form 36 (SF-36). This will be administered by a blinded research nurse.

1. *Postoperative length of stay*

Postoperative length of stay will be measured in days, with the day of the surgery serving as postoperative day 0. Each subsequent morning the patient remains admitted to hospital will serve as an additional day that will contribute to the cumulative postoperative length of stay measure. This measure will only pertain to the admission following the index operation.

**References**

1. McKechnie T, Lee Y, Doumouras A, Parpia S, Bhandari M, Eskicioglu C. The Impact of Very Low Energy Diets Prior to Bariatric Surgery on Postoperative Morbidity: A Systematic Review and Meta-Analysis. Published online 2023.

2. Borchardt RA, Tzizik D. Update on surgical site infections: The new CDC guidelines. *JAAPA (Montvale, NJ)*. 2018;31(4):52-54. doi:10.1097/01.JAA.0000531052.82007.42

3. Rahbari NN, Weitz J, Hohenberger W, et al. Definition and grading of anastomotic leakage following anterior resection of the rectum: A proposal by the International Study Group of Rectal Cancer. *Surgery*. 2010;147(3):339-351. doi:10.1016/j.surg.2009.10.012

4. Regmi S, Bearrick E, Hannah PeterTF, Sathianathen N, Kalapara A, Konety B. Drain fluid creatinine-to-serum creatinine ratio as an initial test to detect urine leakage following cystectomy: A retrospective study. *Indian Journal of Urology*. 2021;37(2):153. doi:10.4103/iju.IJU_396_20

5. de Lima EL, de Brito MJA, da Cunha JB, et al. The Impact of Surgical Wound Dehiscence on Body Image. *Adv Skin Wound Care*. 2018;31(10):470-477. doi:10.1097/01.ASW.0000544616.95630.ac

6. Riou JP, Cohen JR, Johnson H. Factors influencing wound dehiscence. *Am J Surg*. 1992;163(3):324-330. doi:10.1016/0002-9610(92)90014-i

7. Qiu D, Wang X, Wang X, Jiao Y, Li Y, Jiang D. Risk factors for necrosis of skin flap-like wounds after ED debridement and suture. *Am J Emerg Med*. 2019;37(5):828-831. doi:10.1016/j.ajem.2018.07.049

8. Arebro J, Palmgren B. Postsurgical pyoderma gangrenosum and flap necrosis in a head and neck cancer patient following neck dissection. *Clin Case Rep*. 2020;8(7):1121-1125. doi:10.1002/ccr3.2828

9. Mcnally M, Sousa R, Wouthuyzen-Bakker M, et al. Cite this article. *Bone Joint J*. 2021;103(1):18-25. doi:10.1302/0301-620X.103B1

10. Vather R, Trivedi S, Bissett I. Defining Postoperative Ileus: Results of a Systematic Review and Global Survey. *Journal of Gastrointestinal Surgery*. 2013;17(5):962-972. doi:10.1007/s11605-013-2148-y

11. Baker ML, Williams RN, Nightingale JMD. Causes and management of a high-output stoma. *Colorectal disease*. 2011;13(2):191-197. doi:10.1111/j.1463-1318.2009.02107.x

12. Nightingale J. How to manage a high-output stoma. *Frontline Gastroenterol*. 2022;13(2):140-151. doi:10.1136/flgastro-2018-101108

13. Wente MN, Bassi C, Dervenis C, et al. Delayed gastric emptying (DGE) after pancreatic surgery: A suggested definition by the International Study Group of Pancreatic Surgery (ISGPS). *Surgery*. 2007;142(5):761-768. doi:10.1016/j.surg.2007.05.005

14. Pierre S, Whelan R. Nausea and vomiting after surgery. *Continuing Education in Anaesthesia Critical Care & Pain*. 2013;13(1):28-32. doi:10.1093/bjaceaccp/mks046

15. Patel DN, Felder SI, Luu M, Daskivich TJ, Zaghiyan KN, Fleshner P. Early urinary catheter removal following pelvic colorectal surgery: A prospective, randomized, noninferiority trial. *Dis Colon Rectum*. 2018;61(10):1180-1186. doi:10.1097/DCR.0000000000001206

16. de Boer HD, Detriche O, Forget P. Opioid-related side effects: Postoperative ileus, urinary retention, nausea and vomiting, and shivering. A review of the literature. *Best Pract Res Clin Anaesthesiol*. 2017;31(4):499-504. doi:10.1016/j.bpa.2017.07.002

17. Flores-Mireles AL, Walker JN, Caparon M, Hultgren SJ. Urinary tract infections: Epidemiology, mechanisms of infection and treatment options. *Nat Rev Microbiol*. 2015;13(5):269-284. doi:10.1038/nrmicro3432

18. Thomas ME, Blaine C, Dawnay A, et al. The definition of acute kidney injury and its use in practice. *Kidney Int*. 2015;87(1):62-73. doi:10.1038/ki.2014.328

19. Ray K, Bodenham A, Paramasivam E. Pulmonary atelectasis in anaesthesia and critical care. *Continuing Education in Anaesthesia Critical Care & Pain*. 2014;14(5):236-245. doi:10.1093/bjaceaccp/mkt064

20. Ottosen J, Evans H. Pneumonia. *Surgical Clinics of North America*. 2014;94(6):1305-1317. doi:10.1016/j.suc.2014.09.001

21. MacDuff A, Arnold A, Harvey J. Management of spontaneous pneumothorax: British Thoracic Society pleural disease guideline 2010. *Thorax*. 2010;65(Suppl 2):ii18-ii31. doi:10.1136/thx.2010.136986

22. Zeiler J, Idell S, Norwood S, Cook A. Hemothorax: A Review of the Literature. *Clin Pulm Med*. 2020;27(1):1-12. doi:10.1097/CPM.0000000000000343

23. Stratton S. Acute Respiratory Failure. BMJ Best Practices. Published 2023. Accessed August 8, 2023. https://bestpractice.bmj.com/topics/en-us/853

24. Bartlett RH, Morris AH, Fairley HB, Hirsch R, O’Connor N, Pontoppidan H. A Prospective Study of Acute Hypoxic Respiratory Failure. *Chest*. 1986;89(5):684-689. doi:10.1378/chest.89.5.684

25. Kearon C, Akl EA, Ornelas J, et al. Antithrombotic Therapy for VTE Disease. *Chest*. 2016;149(2):315-352. doi:10.1016/j.chest.2015.11.026

26. Devereaux PJ, Szczeklik W. Myocardial injury after non-cardiac surgery: Diagnosis and management. *Eur Heart J*. 2020;41(32):3083-3091. doi:10.1093/eurheartj/ehz301

27. Thygesen K, Alpert JS, White HD. Universal Definition of Myocardial Infarction. *Circulation*. 2007;116(22):2634-2653. doi:10.1161/CIRCULATIONAHA.107.187397

28. Headache Classification Committee of the International Headache Society (IHS). The International Classification of Headache Disorders, 3rd edition. *Cephalalgia*. 2018;38(1):1-211. doi:10.1177/0333102417738202

29. Sacco RL, Kasner SE, Broderick JP, et al. An Updated Definition of Stroke for the 21st Century. *Stroke*. 2013;44(7):2064-2089. doi:10.1161/STR.0b013e318296aeca

30. Seymour CW, Liu VX, Iwashyna TJ, et al. Assessment of Clinical Criteria for Sepsis. *JAMA*. 2016;315(8):762. doi:10.1001/jama.2016.0288

31. Parker W, Wagner W. Management of hemorrhage in gynecologic surgery. UpToDate. Published 2022. Accessed August 8, 2023. https://www.uptodate.com/contents/management-of-hemorrhage-in-gynecologic-surgery
