# Supplementary material for: PReoperative very low-Energy diets for obese PAtients undergoing non-bariatric surgery Randomized Evaluation (PREPARE): a protocol for a pilot randomized controlled trial
Source: Pilot Feasibility Stud. 2024 May 21;10:82. doi: 10.1186/s40814-024-01511-6 (PMC11106982; doi:10.1186/s40814-024-01511-6)
Supplement: Supplementary file 3 — Additional file 3. Adverse events. [file 40814_2024_1511_MOESM3_ESM.docx]

**
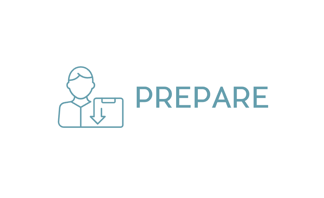
**

**PR**eoperative very low **E**nergy diets for obese **PA**tients undergoing non-bariatric surgery: A **R**andomized **E**valuation

(**PREPARE Pilot)**

*Adverse Events*

Minor Adverse Events

*A.1 Constipation*

For the purposes of this study, constipation will be defined according to the Paris Consensus:^1^

1. Three or less bowel movements per week
2. Large, firm palpable stool in the rectal vault on digital rectal examination

*A.2 Diarrhea*

For the purposes of this study, diarrhea will be defined as:^2^

1. Subjective description of loose stools
2. Increased fecal frequency or urgency

*A.3 Nausea*

For the purposes of this study, nausea will be defined as:^3^

1. Prodromal signs and symptoms that occur prior to emesis
   1. Abdominal discomfort
   2. Anorexia
   3. Anxiety
   4. Loss of interest in ongoing events (Sopite syndrome)

*A.4 Fatigue*

For the purposes of this study, fatigue will be defined as:

1. “Subjective, unpleasant symptom which incorporates total body feelings ranging from tiredness to exhaustion creating an unrelenting overall condition which interferes with individuals’ ability to function to their normal capacity.”^4^

*A.5 Dizziness*

For the purposes of this study, dizziness will be defined as:

1. The subjective feeling of “abnormal sensations relating to perception of the body’s relationship to space.”^5^

*A.6 Headache*

For the purposes of this study, headache will be defined as primary headache disorders, which include:^6^

1. Migraine
2. Tension-type headache

*A.7 Alopecia*

For the purposes of this study, alopecia will be defined as:^7^

1. Development of well demarcated borders of present and absent hair on the scalp

Serious Adverse Events

*A.1 Acute kidney injury*

For the purposes of this study, acute kidney injury will be defined as the presence of at least one of the following three:^8^

1. Urine output of less than 0.5mL/kg/h for more than eight consecutive hours
2. A 50-99% rise in baseline creatinine within a period of seven days
3. A decrease in eGFR of more than 25% within a period of seven days

*A.2 Electrolyte disturbances*

For the purposes of this study, electrolyte disturbance will be defined as any of the following disturbances plus clinical their associated clinical manifestations (not described in the present manual):^9^

1. Serum sodium of less than 136mEq/L or greater than 146mEq/L
2. Serum chloride of less than 96mmol/L or greater than 106mmol/L
3. Serum potassium of less than 3.5mEq/L or greater than 4.5mEq/L
4. Serum magnesium of less than 1.3mEql/L or greater than 2.1mEq/L
5. Serum phosphorus of less than 1.0mmol/L or greater than 1.5mmol/L
6. Serum calcium of less than 9.0mg/dL or greater than 11.0mg/dL

*A.3 Cardiac arrhythmias*

For the purposes of this study, cardiac arrhythmias will be defined as a sustained abnormal myocardial depolarization abnormality recorded via any of the following:^10^

1. ECG
2. Holter monitor
3. Echocardiography
4. Cardiac stress testing (pharmacologic or exercise)

*A.4 Symptomatic Cholelithiasis*

For the purposes of this study, symptomatic cholelithiasis will be defined as:^11^

1. Recurrent post-prandial right upper quadrant and/or epigastric pain
2. Radiographic findings in keeping with cholelithiasis

*A.5 Pancreatitis*

For the purposes of this study, pancreatitis will be defined as the presence of at least two of the following three:^12^

1. Lipase elevated more than three times the upper limit of normal
2. Radiographic findings in keeping with acute pancreatitis
3. Tenderness to palpation in the epigastrium

*A.6 Pyelonephritis*

For the purposes of this study, pyelonephritis will be defined as:^13^

1. Tenderness to palpation of the flank
2. Urinalysis showing pyuria and/or bacteriuria

*A.7 Gout*

For the purposes of this study, gout will be defined as presence of at least two of the following four:^14^

1. Abrupt onset, painful joint swelling
2. Presence of tophi
3. Serum uric acid levels greater than 7mg/dL in males and 6mg/dL in females
4. Presence of urate crystals on synovial fluid analysis

**References**

1. Benninga M, Candy DCA, Catto-Smith AG, et al. The Paris Consensus on Childhood Constipation Terminology (PACCT) Group. *J Pediatr Gastroenterol Nutr*. 2005;40(3):273-275. doi:10.1097/01.MPG.0000158071.24327.88

2. Schiller LR, Pardi DS, Sellin JH. Chronic Diarrhea: Diagnosis and Management. *Clinical Gastroenterology and Hepatology*. 2017;15(2):182-193.e3. doi:10.1016/j.cgh.2016.07.028

3. Balaban CD, Yates BJ. What is nausea? A historical analysis of changing views. *Auton Neurosci*. 2017;202:5-17. doi:10.1016/j.autneu.2016.07.003

4. Ream E, Richardson A. Fatigue: a concept analysis. *Int J Nurs Stud*. 1996;33(5):519-529. doi:10.1016/0020-7489(96)00004-1

5. Sloane PD, Coeytaux RR, Beck RS, Dallara J. *Dizziness: State of the Science*.; 2001. https://annals.org

6. Pellegrino ABW, Davis-Martin RE, Houle TT, Turner DP, Smitherman TA. Perceived triggers of primary headache disorders: A meta-analysis. *Cephalalgia*. 2018;38(6):1188-1198. doi:10.1177/0333102417727535

7. Olsen E, Hordinsky M, McDonald-Hull S, et al. Alopecia areata investigational assessment guidelines*. *J Am Acad Dermatol*. 1999;40(2):242-246. doi:10.1016/S0190-9622(99)70195-7

8. Thomas ME, Blaine C, Dawnay A, et al. The definition of acute kidney injury and its use in practice. *Kidney Int*. 2015;87(1):62-73. doi:10.1038/ki.2014.328

9. Nardone R, Brigo F, Trinka E. Acute symptomatic seizures caused by electrolyte disturbances. *Journal of Clinical Neurology (Korea)*. 2016;12(1):21-33. doi:10.3988/jcn.2016.12.1.21

10. Fu D guan. Cardiac Arrhythmias: Diagnosis, Symptoms, and Treatments. *Cell Biochem Biophys*. 2015;73(2):291-296. doi:10.1007/s12013-015-0626-4

11. SCHIRMER BD, EDGE SB, DIX J, HYSER MJ, HANKS JB, JONES RS. Laparoscopie Cholecystectomy Treatment of Choice for Symptomatic Cholelithiasis. *Ann Surg*. 1991;213(6):665-679. doi:10.1097/00000658-199106000-00018

12. Kiriyama S, Gabata T, Takada T, et al. New diagnostic criteria of acute pancreatitis. *J Hepatobiliary Pancreat Sci*. 2010;17(1):24-36. doi:10.1007/s00534-009-0214-3

13. Johnson JR, Russo TA. Acute Pyelonephritis in Adults. *New England Journal of Medicine*. 2018;378(1):48-59. doi:10.1056/nejmcp1702758

14. Malik A, Schumacher HR, Dinnella JE, Clayburne GM. Clinical diagnostic criteria for gout: Comparsion with the gold standard of synovial fluid crystal analysis. *Journal of Clinical Rheumatology*. 2009;15(1):22-24. doi:10.1097/RHU.0b013e3181945b79
