# Supplementary material for: PReoperative very low-Energy diets for obese PAtients undergoing non-bariatric surgery Randomized Evaluation (PREPARE): a protocol for a pilot randomized controlled trial
Source: Pilot Feasibility Stud. 2024 May 21;10:82. doi: 10.1186/s40814-024-01511-6 (PMC11106982; doi:10.1186/s40814-024-01511-6)
Supplement: Supplementary file 4 — Additional file 4. Full trial power calculation. [file 40814_2024_1511_MOESM4_ESM.docx]

**
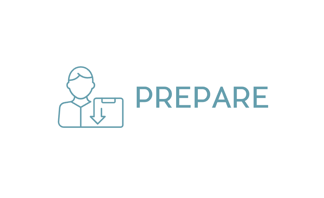
**

**PR**eoperative very low **E**nergy diets for obese **PA**tients undergoing non-bariatric surgery: A **R**andomized **E**valuation

(**PREPARE Pilot)**

*Full Trial Power Calculation*

The primary efficacy endpoint of this fully powered RCT will be 30-day postoperative morbidity. Postoperative morbidity varies widely across specific major intra-abdominal surgeries. At our local institutions, 30-day postoperative morbidity for colorectal surgery approximates 40% and 30-day postoperative morbidity for urologic surgery approximates 10%.^1,2^ Among the greater body of literature pertaining to 30-day postoperative morbidity following major non-bariatric surgery, incidence can range from 5% for some general surgery procedures to 55% for thoracic surgery.^3,4^ The range of 30-day postoperative morbidity, along with 95% confidence intervals, for a variety of operations based on a review of pertinent database studies and systematic reviews can be seen here:

| **Study** | **Type of Study** | **N** | **Point Estimate** | **95% CI** |
| --- | --- | --- | --- | --- |
| *Colorectal surgery* | | | | |
| Richards et al. (2010)^5^ | Systematic review | 768 | 36.0% | 32.7-39.5% |
| McKechnie et al. (2023)^6^ | Database study | 1,174 | 25.6% | 23.6-28.6% |
| *Foregut surgery* | | | |  |
| Shimada et al. (2017)^7^ | Systematic review | 4,604 | 19.0% | 17.9-20.2% |
| Norero et al. (2017)^8^ | Database study | 1,066 | 31.4% | 28.7-34.3% |
| *Hepatobiliary surgery* | | | |  |
| Simmonds et al. (2006)^9^ | Systematic review | 6,529 | 20.1% | 19.1-21.1% |
| Chan et al. (2018)^4^ | Systematic review | 1,030 | 43.1% | 40.1-46.1% |
| *Hernia/abdominal wall surgery* | | | | |
| Joseph et al. (2020)^10^ | Database study | 70,339 | 14.1% | 13.9-14.4% |
| Fischer et al. (2014)^11^ | Database study | 1,706 | 13.4% | 11.8-15.1% |
| *General Surgery* | | | | |
| Fagenson et al. (2021)^12^ | Database study | 6,898 | 4.4% | 3.9-4.9% |
| Tustumi et al. (2018)^13^ | Systematic review | 27,076 | 7.0% | 6.7-7.3% |
| *Urology* | | | | |
| Khalil et al. (2019)^14^ | Database study | 1,290 | 4.7% | 3.7-6.1% |
| Maibom et al. (2021)^15^ | Systematic review | 19,160 | 39.0% | 27.3-49.0% |
| Zakaria et al. (2014)^16^ | Database study | 2,778 | 30.6% | 28.9-32.3% |
| *Gynecology* | | | | |
| Dowdy et al. (2012) | Database study | 1,415 | 28.1% | 25.8-30.5% |
| Long et al. (2014)^17^ | Systematic review | 636 | 16.0% | 13.4-19.0% |
| *Orthopedic Surgery* | | | | |
| Schoenfeld et al. (2013) ^18^ | Database study | 11,004 | 26.0% | 25.2-26.8% |
| Li et al. (2017)^19^ | Database study | 103,290 | 17.2% | 17.0-17.5% |
| *Cardiac Surgery* | | | | |
| Syraka et al. (2007)^20^ | Database study | 10,888 | 18.3% | 17.5-19.0% |
| Turnan et al. (1992)^21^ | Database study | 3,156 | 22.2% | 20.7-23.7% |
| *Thoracic Surgery* | | | | |
| Rueth et al. (2012) | Database study | 4,171 | 55.8% | 54.3-57.3% |
| Hu et al. (2020) | Systematic review | 4,899 | 28.5% | 27.2-29.7% |
| *Otolaryngology* | | | | |
| Van Slycke et al. (2021)^22^ | Database study | 1,043 | 7.5% | 5.9-9.3% |
| Schwam et al. (2015)^23^ | Database study | 408 | 21.3% | 17.4-25.6% |
| *Plastic Surgery* | | | | |
| Sebai et al. (2019)^24^ | Systematic review | 822 | 10.8% | 8.2-12.5% |

Given this variability, we feel as though a mean value of 20-25% risk of 30-day postoperative morbidity for our study population is reasonable. The mean prevalence of 30-day postoperative morbidity across all of the above database studies and systematic reviews is approximately 22%. As such, this is the baseline risk on which we will base our sample size calculation.

Our previous systematic review and meta-analysis failed to identify any significant difference in 30-day postoperative morbidity in the four studies that compared patients receiving and not receiving VLED prior to major abdominal surgery. None of the included studies were adequately powered, however, and all reported lower than expected baseline risk of 30-day postoperative morbidity. A subsequent population-level retrospective cohort study performed by our research group which evaluated preoperative weight loss with bariatric surgery prior to undergoing colorectal surgery demonstrated a 32% relative risk reduction in 30-day postoperative morbidity in the bariatric surgery group. As such, we feel as though an expected 30% relative reduction, equating to a 7% absolute reduction in 30-day postoperative morbidity in our full-powered RCT in the patients receiving preoperative VLED is reasonable. We searched previous literature to attempt to compare this with any previous estimation of a minimally clinical important difference (MCID) in this patient population. There was no previous data pertaining to this. As such, we confirmed with local colorectal, urologic, and general surgeons that a 7% absolute risk reduction in 30-day postoperative morbidity in patients undergoing major intra-abdominal surgery, would be a reasonable MCID.

Therefore, assuming an expected proportion of 30-day postoperative morbidity of 0.22 in the control group and 0.15 in the intervention group, a power of 80%, an alpha of 0.05, two-sided tests, 1:1 allocation, and the use of a chi-square test, a sample size of 964 patients (482 patients per arm) would be required.^25^ In order to accommodate for lack of compliance, loss to follow-up, and to provide a “fragility buffer”, we inflated our sample size calculation by 20% to plan for a total sample size of 1,158 patients (579 patients per arm).^26^

**References**

1. McKechnie T, Ramji K, Kruse C, et al. Posterior mesorectal thickness as a predictor of increased operative time in rectal cancer surgery: a retrospective cohort study. *Surg Endosc*. 2022;36(5):3520-3532. doi:10.1007/s00464-021-08674-w

2. Millan B, Cassim R, Uy M, Bay B, Shayegan B. First Canadian experience with same-day discharge after robot-assisted radical prostatectomy. *Canadian Urological Association Journal*. 2022;17(2):1-11. doi:10.5489/cuaj.7914

3. Fagenson AM, Powers BD, Zorbas KA, et al. Frailty Predicts Morbidity and Mortality After Laparoscopic Cholecystectomy for Acute Cholecystitis: An ACS-NSQIP Cohort Analysis. *Journal of Gastrointestinal Surgery*. 2021;25:932-940. doi:10.1007/s11605-020-04570-1/Published

4. Chan J, Perini M, Fink M, Nikfarjam M. The outcomes of central hepatectomy versus extended hepatectomy: a systematic review and meta-analysis. *HPB*. 2018;20(6):487-496. doi:10.1016/j.hpb.2017.12.008

5. Richards CH, Leitch FE, Horgan PG, McMillan DC. A Systematic Review of POSSUM and its Related Models as Predictors of Post-operative Mortality and Morbidity in Patients Undergoing Surgery for Colorectal Cancer. *Journal of Gastrointestinal Surgery*. 2010;14(10):1511-1520. doi:10.1007/s11605-010-1333-5

6. McKechnie T, Lee Y, Hong D, et al. The Impact of a History of Bariatric Surgery for Weight Reduction Prior to Surgery for Colorectal Cancer: Analysis of the National Inpatient Sample 2015-2019 .

7. Shimada H, Fukagawa T, Haga Y, Oba K. Does postoperative morbidity worsen the oncological outcome after radical surgery for gastrointestinal cancers? A systematic review of the literature. *Ann Gastroenterol Surg*. 2017;1(1):11-23. doi:10.1002/ags3.12002

8. Norero E, Vega EA, Diaz C, et al. Improvement in postoperative mortality in elective gastrectomy for gastric cancer: Analysis of predictive factors in 1066 patients from a single centre. *European Journal of Surgical Oncology*. 2017;43(7):1330-1336. doi:10.1016/j.ejso.2017.01.004

9. Simmonds PC, Primrose JN, Colquitt JL, Garden OJ, Poston GJ, Rees M. Surgical resection of hepatic metastases from colorectal cancer: A systematic review of published studies. *Br J Cancer*. 2006;94(7):982-999. doi:10.1038/sj.bjc.6603033

10. Joseph WJ, Cuccolo NG, Baron ME, Chow I, Beers EH. Frailty predicts morbidity, complications, and mortality in patients undergoing complex abdominal wall reconstruction. *Hernia*. 2020;24(2):235-243. doi:10.1007/s10029-019-02047-y

11. Fischer JP, Wink JD, Nelson JA, Kovach SJ. Among 1,706 cases of abdominal wall reconstruction, what factors influence the occurrence of major operative complications? *Surgery (United States)*. 2014;155(2):311-319. doi:10.1016/j.surg.2013.08.014

12. Fagenson AM, Powers BD, Zorbas KA, et al. Frailty Predicts Morbidity and Mortality After Laparoscopic Cholecystectomy for Acute Cholecystitis: An ACS-NSQIP Cohort Analysis. doi:10.1007/s11605-020-04570-1/Published

13. Tustumi F, Bernardo WM, Santo MA, Cecconello I. Cholecystectomy in Patients Submitted to Bariatric Procedure: A Systematic Review and Meta-analysis. *Obes Surg*. 2018;28(10):3312-3320. doi:10.1007/s11695-018-3443-1

14. Khalil MI, Bhandari NR, Payakachat N, Davis R, Raheem OA, Kamel MH. Perioperative mortality and morbidity of outpatient versus inpatient robot-assisted radical prostatectomy: A propensity matched analysis. *Urologic Oncology: Seminars and Original Investigations*. 2020;38(1):3.e1-3.e6. doi:10.1016/j.urolonc.2019.07.008

15. Maibom SL, Joensen UN, Poulsen AM, Kehlet H, Brasso K, Røder MA. Short-term morbidity and mortality following radical cystectomy: A systematic review. *BMJ Open*. 2021;11(4). doi:10.1136/bmjopen-2020-043266

16. Zakaria AS, Santos F, Dragomir A, Tanguay S, Kassouf W, Aprikian AG. Postoperative mortality and complications after radical cystectomy for bladder cancer in Quebec: A population-based analysis during the years 2000-2009. *Canadian Urological Association Journal*. 2014;8(7-8):259-267. doi:10.5489/cuaj.1997

17. Long Y, Yao DS, Pan XW, Ou TY. Clinical efficacy and safety of nerve-sparing radical hysterectomy for cervical cancer: A systematic review and meta-analysis. *PLoS One*. 2014;9(4). doi:10.1371/journal.pone.0094116

18. Schoenfeld AJ, Serrano JA, Waterman BR, Bader JO, Belmont PJ. The impact of resident involvement on post-operative morbidity and mortality following orthopaedic procedures: A study of 43,343 cases. *Arch Orthop Trauma Surg*. 2013;133(11):1483-1491. doi:10.1007/s00402-013-1841-3

19. Li X, Veltre DR, Cusano A, et al. Insurance status affects postoperative morbidity and complication rate after shoulder arthroplasty. *J Shoulder Elbow Surg*. 2017;26(8):1423-1431. doi:10.1016/j.jse.2016.12.071

20. Syrakas CA, Neumaier-Prauser P, Angelis I, Kiask T, Kemkes BM, Gansera B. Is extreme obesity a risk factor for increased in-hospital mortality and postoperative morbidity after cardiac surgery? Results of 2251 obese patients with BMI of 30 to 50. *Thoracic and Cardiovascular Surgeon*. 2007;55(8):491-493. doi:10.1055/s-2007-965599

21. Tuman KJ, Robert FCCP;, Mccarthy J, March R, Najafi H, Lvankovich ACP; D. *Morbidity and Duration of ICU Stay after Cardiac Surgery* A Model for Preoperative Risk Assessment*.

22. van Slycke S, van den Heede K, Bruggeman N, Vermeersch H, Brusselaers N. Risk factors for postoperative morbidity after thyroid surgery in a PROSPECTIVE cohort of 1500 patients. *International Journal of Surgery*. 2021;88. doi:10.1016/j.ijsu.2021.105922

23. Schwam ZG, Sosa JA, Roman S, Judson BL. Complications and mortality following surgery for oral cavity cancer: Analysis of 408 cases. *Laryngoscope*. 2015;125(8):1869-1873. doi:10.1002/lary.25328

24. Sebai ME, Siotos C, Payne RM, et al. Enhanced Recovery after Surgery Pathway for Microsurgical Breast Reconstruction: A Systematic Review and Meta-Analysis. In: *Plastic and Reconstructive Surgery*. Vol 143. Lippincott Williams and Wilkins; 2019:655-666. doi:10.1097/PRS.0000000000005300

25. Hulley S, Cummings S, Browner W, Grady D, Newman T. *Designing Clinical Research: An Epidemiologic Approach*. 4th ed. Lippincott Williams & Wilkins; 2013.

26. Ekhtiari S, Gazendam AM, Nucci NW, Kruse CC, Bhandari M. The Fragility of Statistically Significant Findings From Randomized Controlled Trials in Hip and Knee Arthroplasty. *Journal of Arthroplasty*. 2021;36(6):2211-2218.e1. doi:10.1016/j.arth.2020.12.015
